# Elevated expression of NFE2L3 promotes the development of gastric cancer through epithelial-mesenchymal transformation

**DOI:** 10.1080/21655979.2021.2005915

**Published:** 2021-12-13

**Authors:** Xiaodong Wang, Yaxian Li, Ziqing Fang, Yongxiang Li

**Affiliations:** Department of General Surgery, The First Affiliated Hospital of Anhui Medical University, Hefei, People’s Republic of China

**Keywords:** Gastric cancer, EMT, NFE2L3, bioinformatics

## Abstract

Gastric cancer (GC) is a malignant tumor with high mortality, but research on its molecular mechanisms remain limited. This study is the first to explore the biological role of nuclear factor NFE2L3 (nuclear factor, erythroid 2 like 3) in GC. We used Western blot and RT–qPCR to detect gene expression at the protein or mRNA level. Short hairpin RNA (shRNA) transfection was used to inhibit NFE2L3 expression. CCK-8 and colony formation assays were used to detect cell proliferation. Cell migration, invasion, cell cycle and apoptosis were detected by Transwell assays and flow cytometry. The results showed that NFE2L3 was highly expressed in gastric cancer tissues and promoted gastric cancer cell proliferation and metastasis. Inhibiting NFE2L3 expression blocks the cell cycle and increases the proportion of apoptotic cells, whereas NFE2L3 expression promotes the epithelial-mesenchymal transformation (EMT) process. In summary, NFE2L3 is highly expressed in gastric cancer and promotes gastric cancer cell proliferation and metastasis and the EMT process.

## 1. Introduction

GC is one of the most common gastrointestinal malignancies, and its morbidity (5.6%) and mortality (7.7%) ranks fifth and fourth among all cancers, respectively [1]. At present, surgical resection, chemotherapy and radiotherapy remain the main modalities for the treatment of GC [2], but the efficacy of these treatments in patients with advanced GC remain unsatisfactory [3,4]. Due to the lack of effective diagnostic means, GC patients are often in the advanced stage once discovered. Therefore, the identification of effective gene targets is an important strategy for future diagnosis and prolonging the survival time of GC patients.

Nuclear factor erythroid 2-like 3 (NFE2L3), which is also known as NRF3, is a transcription factor that is a member of the cap ‘n’ collar (CNC) basic-region leucine zipper family and was first identified in 1999 [5]. Although the CNC family has been reported to be closely related to the occurrence of tumors, especially its homologous NFE2L2, which has received extensive attention, studies on NFE2L3 have been delayed due to the absence of significant phenotypic differences in NFE2L3 knockout mice [6,7]. In recent years, with in-depth research on CNC family members, NFE2L3 has gradually come into our field of vision. Similar to NFE2L2, NFE2L3 is also involved in oxidative stress in the body [8–11].

Deletion of the NFE2L3 gene made mice more susceptible to lymphoma, suggesting that the transcription factor may have a protective role in the blood system [12]. The same protective effect also occurred in breast cancer patients. Specifically, when NFE2L3 was inhibited in breast cancer tissues, the proliferation and metastasis of breast cancer cells was reduced [13]. Moreover, the NFE2L3 gene has been shown to promote cancer in additional studies. Increased NFE2L3 expression predicts a poor prognosis in patients with pancreatic cancer [14]. NFE2L3 knockdown can induce apoptosis and inhibit the formation and proliferation of hepatocellular carcinoma cells [15,16]. In colorectal cancer, NFE2L3 expression in tumor tissues was significantly higher than that in paired paracancerous tissues, and inhibition of NFE2L3 expression also caused tumor cell proliferation to arrest in the G0/G1 phase [17]. An increasing number of studies have shown the role of the NFE2L3 gene in tumors, which may be a key regulatory factor in the process of tumorigenesis. However, the role of the NFE2L3 gene in GC remains uncharacterized, and further exploration is needed.

In our study, we explored NFE2L3 expression in gastric cancer for the first time, constructed an NFE2L3 knockdown cell line to verify the effect of NFE2L3 on gastric cancer cell proliferation and metastasis, and assessed the cell cycle and apoptosis.

## 2. Materials and methods

### 2.1 Online database

To explore the expression of NFE2L3 in tumors, we downloaded tumor RNA-seq data from The Cancer Genome Atlas (TCGA) database (https://portal.gdc.cancer.gov/) and Gene Expression Omnibus (GEO) database (https://www.ncbi.nlm.nih.gov/gds). The relationship between NFE2L3 gene expression and the survival time of GC patients was predicted based on the Kaplan–Meier plotter (http://kmplot.com) database. Other NFE2L3-related genes were predicted from the STRING (https://string-db.org/) website. The association between NFE2L3 and immune cell infiltration was predicted using the TIMER 2.0 (http://timer.comp-genomics.org) website.

### 2.2 Immunohistochemical (IHC)

The wax blocks containing human GC tissue were continuously sliced, dewaxed with xylene solution, and dehydrated in gradient ethanol. Next, after inactivating endogenous peroxidase, the slices were placed in citrate buffer (pH = 6.0) and boiled for 15 min in a microwave oven for antigen retrieval. The slices were bound to NFE2L3 (Affinity, China, 1:200) antibody, incubated overnight in a refrigerator at 4°C, and then placed at room temperature for 20 min to allow secondary antibody binding. Finally, diaminobenzidine tetrahydrochloride (DAB) (Proteintech, Wuhan, China) working solution was added for visualization, and the nucleus was stained with hematoxylin. Our specimens were obtained from surgically resected GC tissue with the consent of the patient. Images were captured using an upright light microscope (DM6B, Leica).

### 2.3 Cell culture

The normal gastric epithelial cell line GES-1 and gastric cancer cell lines AGS, SGC-7901, MGC-803 and BGC-823 were purchased from GeneChem (Shanghai, China). Cells were cultured in RPMI-1640 (Corning, USA) medium with 10% fetal bovine serum (Gibco, USA), 1% penicillin (100 U/ml) and 1% streptomycin (100 μg/ml) (HyClone, USA). All the cells were cultured in our configured medium, and the transfected cells were cultured in complete medium supplemented with puromycin (HyClone, USA). All the cells were cultured in a humidified incubator at 37°C with 95% air and 5% CO_2_.

### 2.4 Immunofluorescence

To assess the cellular localization of the protein, SGC-7901 cells were inoculated on cell slides in a 35-mm petri dish for experiments. After permeabilization with 0.5% Triton X-100 for 20 min at room temperature, the membrane was sealed with 5% BSA solution. The NFE2L3 antibody (Affinity, China, 1:200) was added to a wet box, and slides were incubated in a refrigerator at 4°C overnight. On the second day, the cells were incubated with fluorescent secondary antibody (Thermo, USA, 1:250) for 60 min at room temperature. The nuclei were stained with DAPI under dark conditions. Images were obtained using an upright light microscope (DM6B, Leica).

### 2.5 Western blot

After washing the cell culture dishes with PBS, M-per protein lysate containing phosphatase and protease inhibitors (BBI Life Sciences Corporation, China) was added to lyse the cells to obtain cell proteins. After gel electrophoresis was performed on an acrylamide gel, the proteins were transferred to PVDF membranes (Millipore, USA) and sealed with 5% skimmed milk. Membranes were incubated with the primary antibody overnight in a refrigerator at 4°C. On the second day, after exposure to a mouse or rabbit secondary antibody for 1 h, chemiluminescence solution was used for detection.

Antibodies for immunoblotting included GAPDH (CST, USA, 1:4000), NFE2L3 (Afffinity, China, 1:500), BAX (Santa-Cruz, China, 1:1000), CDC2 (CST, China, 1:1000), Caspase 3 (Santa-Cruz, China, 1:1000), Bcl2 (Abcam, China, 1:1000), N-cadherin (CST, China, 1:1000), Vimentin (CST, China, 1:1000), Snail (CST, China, 1:1000), and E-cadherin (CST, China, 1:1000).

### 2.6 Cell transfection

Human NFE2L3 shRNA interference lentiviral vector was purchased from GeneChem (Shanghai, China). The NFE2L3 shRNA interference target sequences were sh1:5’-GATAGAAACTTGAGCCGTGAT-3’, sh2:5’-CCGCGTAGACCTAGATCTTTA-3’, and sh3:5’-GGGCATCT CATTGGGAGATAT-3’. According to the manufacturer’s requirements, the cells were inoculated on 24-well plates at a density of 60,000 per well for SGC-7901 cells and 30,000 per well for MGC-803 cells and transfected with lentivirus supernatant (MOI = 10). Culture medium containing puromycin was used for screening. The interference efficiency of NFE2L3 was detected by RT–qPCR and Western blot.

### 2.7 RNA extraction and quantitative real‑time PCR (qRT‑PCR)

Using TRIzol Reagent (Invitrogen, USA), total cellular RNA was extracted according to the manufacturer’s instructions. The extracted RNA was reverse transcribed into cDNA using PrimeScript RT-polymerase (Takara Bio, Dalian, China). Then, the SYBR Green kit was used for qRT-PCR according to the specified reaction conditions. GAPDH served as the internal reference for all of the results, and the 2^−ΔΔCt^ method was used to calculate the relative expression of the gene. The primer sequences were as follows: GAPDH forward 5’-GCA TCC TGG GCT ACACT-3’ and reverse 5’-CAC CAC CCT GTT GCTGT-3’; NFE2L3 forward 5’- CAC AGA TAG AAA CTT GAG CCGT −3’ and reverse 5’- GCG TTT ACG ACA GTT CTGCG −3’. The primer sequences were based on previously published literature [15]. A quantitative real-time PCR instrument (MX3000P, Agilent) was used.

### 2.8 Cell counting kit-8 (CCK-8) assay

The cells were diluted to 30,000/ml and then inoculated into 96-well plates at 100 µL per well. Cell viability was detected every 24 h according to the instructions of the CCK-8 detection kit, and the OD value at 450 nm was determined by using a microplate reader (ELX800, BioTek).

### 2.9 Colony formation assay

The cells were diluted into a cell suspension, and 500 cells were inoculated in a 60-mm cell culture dish and incubated in a temperature box at 37°C for 14 days. The culture medium used was the described configured complete medium containing puromycin. After the cells formed colonies, the petri dishes were washed with PBS, fixed with 4% paraformaldehyde, stained with 0.1% crystal violet, and finally air-dried. Microscopy was used to count the number of cell colonies and compare the differences.

### 2.10 Migration and invasion assays

We used a 24-well plate with a pore diameter of 8 μm to perform cell migration and invasion experiments. In the migration experiment, GC cells were diluted with serum-free RPMI-1640 medium and inoculated in the upper chamber at a density of 5 × 10^4^ cells per well, whereas 600 μL 20% fetal bovine serum was added to the lower chamber. Cells were incubated in an incubator for 24 hours. Next, cells were fixed with 4% paraformaldehyde, stained with 0.1% crystal violet, and counted under 100 X magnification using a microscope. For the invasion experiment, we applied Matrigel (BD Biosciences, USA) to the bottom of the chamber in advance to simulate the biological basement membrane.

### 2.11 Cell cycle analysis by flow cytometry

The cells of the experimental group and control group were collected, washed with PBS, centrifuged to remove the supernatant, and fixed with 70% ethanol at 4°C for 2 hours. The cells were washed with PBS thrice and stained with propidium iodide in a dark environment for 30 min. The proportion of GO/G1 phase, S phase and G2 phase cells in the cell cycle was detected by Cytoflex flow cytometry (Beckman, USA).

### 2.12 Statistical analysis

GraphPad Prism 7.0 (GraphPad Software Inc., USA) was used for graphing. SPSS 25.0 (SPSS Inc., USA) software was used to compare the differences between the treatment group and the control group by non-parametric test. The data are expressed as the mean ± standard deviation (SD). All results were replicated at least thrice. Here, P < 0.05 was considered to be significant with ‘*’, ‘**’, and ‘***’ representing P values less than 0.05, 0.01, and 0.001, respectively.

## 3. Results

### 3.1 NFE2L3 was upregulated in GC tissues and cells

**Figure 1.**
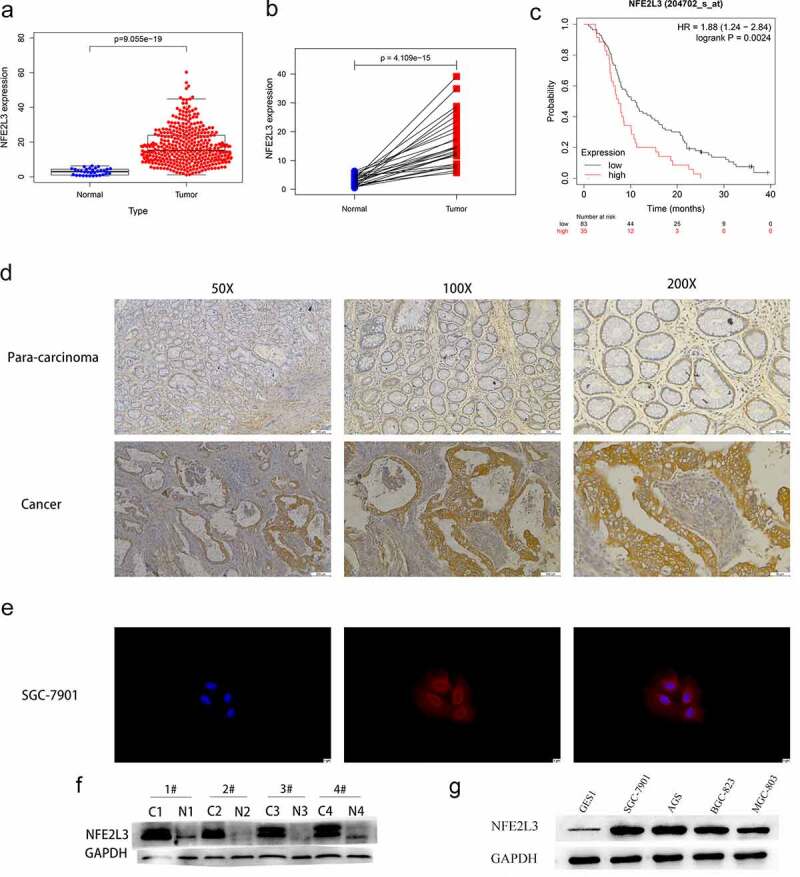
**NFE2L3 is highly expressed in gastric cancer** (a) the expression level of NFE2L3 in GC was increased. (b) Paired sample analysis showed that the expression of NFE2L3 in tumors was still significantly increased. (c) Kaplan-Meier Plotter website predicted that patients with high expression of NFE2L3 had poorer survival time. (d) Immunohistochemical results showed that the expression of NFE2L3 in tumor tissues was higher than that in adjacent tissues. (e) Immunofluorescence showed the localization of NFE2L3 in GC cells, mainly concentrated in the cytoplasm. (f) Western blot assay showed that the expression of NFE2L3 in tumor was significantly higher than that in paracancerous tissues. (g) the expression of NFE2L3 in GC cell lines was higher than that in normal gastric mucosal cell lines

To explore NFE2L3 expression in GC, we downloaded and analyzed RNA-seq data from the GEO database (GSE103236) and TCGA database. As shown in Table 1, NFE2L3 expression in GC was considerably increased compared with that in paracancerous tissues (adjusted P value = 0.01). Next, we used R software to analyze the TCGA data and obtained the same results (Figure 1(a-b)). Patients with high expression of the NFE2L3 gene had shorter survival times (Figure 1(c)). Our immunohistochemical and Western blot experiments further confirmed that NFE2L3 expression was significantly increased in GC tissues (Figure 1(d-f)). Compared with that in GES1 normal gastric mucosal cells, the expression of NFE2L3 in four GC cell lines (SGC7901, AGS, MGC803, and BGC823) was also increased (Figure 1(g)). Based on the immunofluorescence results, we observed that the NFE2L3 gene was mostly located in the cytoplasm (Figure 1(e)).

**Table 1.** GSE103236

Gene.title	Gene.symbol	P.Value	adj.P.Val^a^	logFC^b^
nuclear factor, erythroid 2 like 3	NFE2L3	5.08E-05	0.0140779	1.35179291
integrin subunit beta like 1	ITGBL1	6.59E-02	0.3211426	1.24112061
diaphanous related formin 3	DIAPH3	9.59E-03	0.1418267	1.03553343
solute carrier family 12 member 7	SLC12A7	1.76E-04	0.0239555	0.83344261
RAN binding protein 1	RANBP1	8.92E-04	0.0482718	0.78950276
sperm associated antigen 9	SPAG9	6.11E-02	0.3111831	0.51256638
cell division cycle 42	CDC42	9.01E-02	0.3618538	0.3797353
mitogen-activated protein kinase 14	MAPK14	4.23E-01	0.6718137	0.19319012
oxidation resistance 1	OXR1	8.39E-01	0.9300662	0.04555309
myogenic differentiation 1	MYOD1	4.21E-01	0.6705817	−0.223518
pentatricopeptide repeat domain 2	PTCD2	6.45E-02	0.3182058	−0.4731941
aprataxin and PNKP like factor	APLF	8.84E-02	0.35931	−0.5124112
rhophilin associated tail protein 1	ROPN1	8.27E-02	0.3502687	−0.5912329
hydroxyacyl-CoA dehydrogenase	HADH	9.05E-05	0.0169817	−0.9953331
guanidinoacetate N-methyltransferase	GAMT	1.27E-04	0.0196593	−1.4185967
estrogen receptor 2	ESR2	1.70E-02	0.1857686	−1.5782673

a Adjusted P-value

b Log fold change: logFC represents the ratio of expression between the two groups of samples, and the logarithm with 2 as the base is called logFC.

### 3.2 Knockdown of NFE2L3 Inhibits GC Cell Proliferation and Metastasis

**Figure 2.**
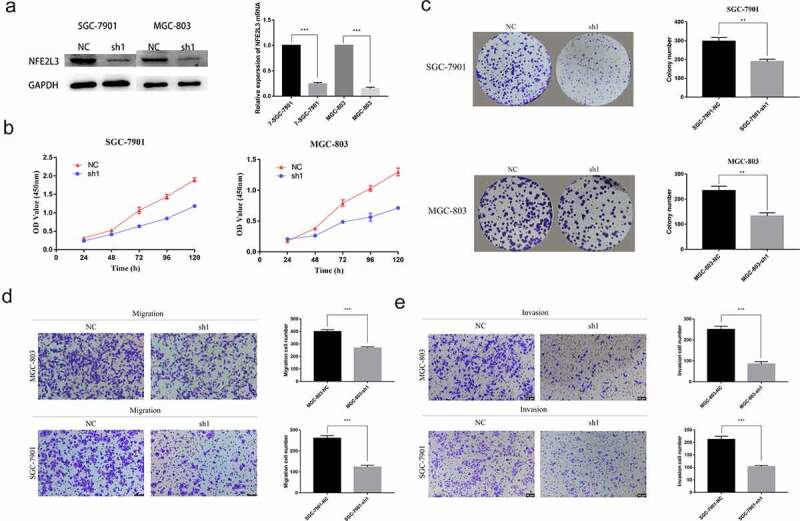
**Silencing NFE2L3 inhibited cell proliferation and metastasis** (a) Western blot and RT-qPCR were used to detect the protein expression and mRNA changes of NFE2L3 in GC cells after lentivirus gene knockout. (b) CCK-8 assay was used to detect the proliferation of SGC-7901 and MGC803 cells at 24, 48, 72, 96 and 120 h after NFE2L3 knockdown. (c) In colony formation assays, NFE2L3 knockdown significantly reduced the colony numbers. (d, e) The migration and invasion ability of the cells silenced by NFE2L3 were significantly decreased (***p < 0:001; **p < 0:01; *p < 0:05)

To assess the role of NFE2L3 in GC cells, we used a short hairpin RNA (shRNA) interference lentiviral vector to knockdown NFE2L3 gene expression. As shown in Figure 2(a), compared with the negative control group, the interference efficiency of shRNA in GC cell lines was greater than 70%. First, we used the CCK-8 method to explore cell proliferation. After measuring OD values at continuous and fixed times, we found that MGC803 and SGC7901 cell proliferation in the knockdown group was lower than that in the control group (Figure 2(b)). This result was consistent with the results of the subsequent plate colony formation experiment. Cell lines with NFE2L3 knockdown exhibited a significantly reduced ability to form colonies (Figure 2(c)). Subsequently, we performed a Transwell assay to examine the effect of the NFE2L3 gene on cell migration and invasion. The results showed that MGC803 and SGC7901 cells with reduced cell migration and invasion exhibited reduced NFE2L3 gene expression (Figure 2(d-e)). The above results confirmed that NFE2L3 plays an important role in GC cell proliferation and metastasis.

### 3.3 Downregulation of NFE2L3 inhibited GC cell growth at the G0/G1 phase and induced GC cell apoptosis

**Figure 3.**
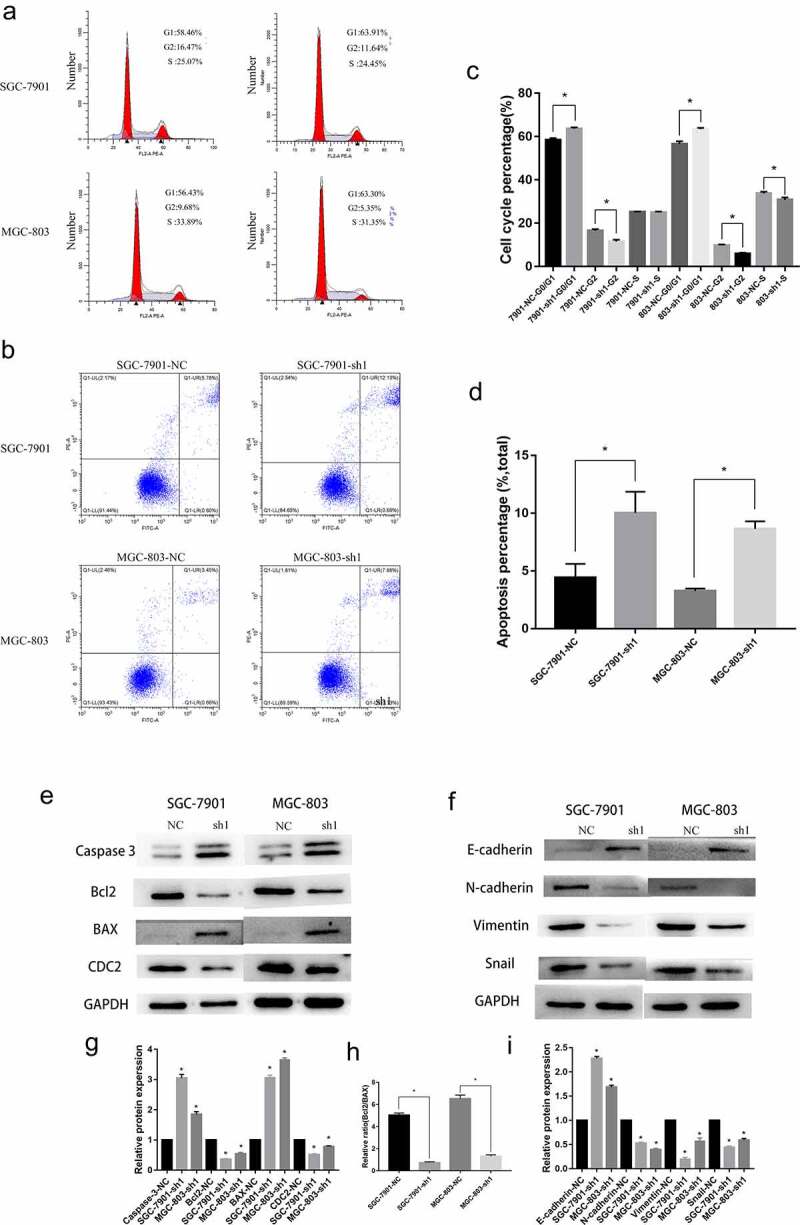
**Knockdown of NFE2L3 arrested cell cycle induced apoptosis and was associated with EMT** (a, c) Flow cytometry was used to detect the changes of cell cycle, and histogram was used to quantify the distribution of cell cycle changes in G1, G2 and S phase. (b, d) Flow cytometry was used to detect the changes of apoptosis, and a column chart was used to show the changes in the proportion of apoptosis. (e, g) Western blot was used to detect the expression of key proteins regulating apoptosis and cycle. (f, i) to detect the expression changes of key proteins during the development of EMT. (h) The changes of the ratio of Bcl-2/BAX protein reflected the changes of apoptosis (***p < 0:001; **p < 0:01; *p < 0:05)

Subsequently, we explored the effects of NFE2L3 on the GC cell cycle and apoptosis. Compared with the control group, GC cells in the NFE2L3 knockdown group showed G0/G1 phase arrest; thus, these cells remain in the G0/G1 phase. Moreover, the percentage of cells in G2 phase decreased significantly (Figure 3(a-b)). In addition, we found that the proportion of apoptotic cells increased with downregulation of NFE2L3, indicating that NFE2L3 may promote the cell cycle and inhibit apoptosis (Figure 3(c-d)). We examined the expression of some proteins related to the cell cycle and apoptosis to further validate the molecular mechanism. As shown in Figure 3(e-g), NFE2L3 knockdown promoted Caspase3 and BAX expression and inhibited Bcl2 and CDC2 protein expression. The ratio changes of Bcl-2/BAX protein were shown in Figure 3(h). Some studies have reported that NFE2L3 is closely related to the EMT process, and we also verified the relationship between the expression of NFE2L3 and EMT-related proteins in GC cells. When NFE2L3 was knocked down, E-cadherin expression was increased, whereas N-cadherin, vimentin and Snail expression was inhibited. This finding is consistent with previously reported results (Figure 3(f-i)). NFE2L3 may be involved in the occurrence of the EMT of tumor cells in vivo.

### 3.4 Prediction of genes interacting with NFE2L3 and GO enrichment analysis

**Figure 4.**
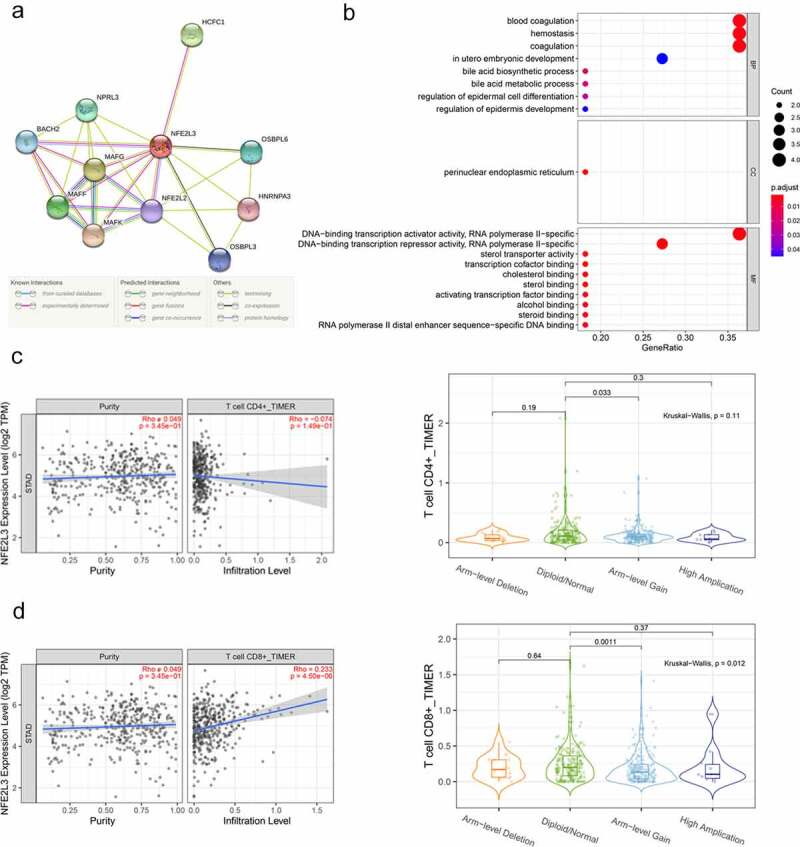
**Establishment of Protein-Protein Interaction (PPI) Network and GO functional enrichment analysis** (a) The genes related to NFE2L3 were predicted using the STRING website and plotted the PPI network. The lines with different colors indicated different prediction relationships, and the specific relationships were explained below the figure. (b) Use NFE2L3 and related genes to draw the enrichment analysis diagram of GO function. The size of the circle represents the level of correlation, the color change means the size of P value. (c,d) Firstly, the scattered plot of tumor purity on the left is used for correction, and the correlation analysis between NFE2L3 and CD4 + T cells and CD8 + T cells is carried out. The vertical axis is the expression of NFE2L3, the horizontal axis is the infiltration level of T cells, and the upper right is the correlation coefficient and P value. The violin diagram shows the difference in T cell expression with different copy number of NFE2L3 gene, P < 0.05 indicates a statistical difference

The associated proteins coordinate with each other to perform their physiological functions in vivo, so we used the STRING website to predict the proteins interacting with NFE2L3 and construct a protein–protein interaction (PPI) network to further explore the biological functions of these proteins. As shown in Figure 4(a), a total of 10 genes were predicted to exhibit a strong association with NFE2L3, and the lines between proteins represented different predicted relationships as detailed in the line annotations. We also conducted GO enrichment analysis on these proteins using R software, and the results are shown in Figure 4(b). A series of NFE2L3-related proteins were significantly enriched in the regulation of epidermal cell differentiation, regulation of epidermal development, DNA-binding transcription activator RNA polymerase II-specific activity, DNA binding repressor RNA polymerase II-specific activity, blood coagulation, hemostasis, and other biological functions.

Additionally, we predicted the association between NFE2L3 and immune cells using the TIMER2.0 website. After tumor purity correction, we found that NFE2L3 expression levels were negatively correlated with the infiltration level of CD4 T cells and significantly positively correlated with the infiltration level of CD8 T cells. Due to genomic instability, cancer cells usually harbor a large number of copy number variations, which play a key role in the occurrence and development of tumors [18]. Then, based on the differences in NFE2L3 gene copy numbers in the tumor, we divided GC patients into four groups, including ‘arm-level deletion’, ‘diploid/normal’, ‘arm-level gain’, and ‘high amplification’, which indicate the loss of an arm-level somatic copy, the normal number of copies, obtaining an arm-level Somatic copy, and many copies, respectively. A significant difference in the infiltration degree of CD4 and CD8 T cells were noted in tumors from the ‘diploid/normal’ and ‘ARM-level gain’ groups. These studies reveal some of the potential effects of NFE2L3 on tumor immunity (Figure 4(c-d)).

## 4. Discussion

Surgical resection remains the first choice for patients with GC. Although diagnostic methods are constantly being updated, however, most patients are often in an advanced stage at the time of initial diagnosis [19], which is also an important reason explaining the high rate of GC recurrence and distant metastasis [20]. These features represent significant difficulties in the treatment of GC. Therefore, the search for new molecular markers represents the key to diagnosis and treatment. Gene transcriptional regulation, epigenetic changes and environmental changes lead to damage to normal cell biological functions and subsequently promote the occurrence of tumors [21–23]. Among them, oxidative stress is closely related to the occurrence of a variety of tumors [24,25], and NFE2L3 also plays an important role in oxidative stress [9]. NFE2L3 plays an important role in other tumors, but studies on NFE2L3 in GC remain limited.

Based on the development of bioinformatics, transcriptional spectrum data from databases have been used as a new research method to identify the key genes regulating tumors and expand the research results beyond experimental results [26]. In this study, using the expression profiles reported in TCGA and GEO, we found that NFE2L3 expression in GC tissues was significantly higher than that in paracancerous tissues. We confirmed that NFE2L3 expression is upregulated in GC by immunohistochemistry and Western blot detection of GC cells and tissues. These findings suggest that NFE2L3 may be a potential oncogene that regulates the occurrence and development of GC.

In the subsequent functional experiment, we constructed two NFE2L3 knockdown cell lines, and the results showed that the growth rate of these GC cells was significantly inhibited. In the colony formation experiment, the number of colonies formed by GC cells decreased in the knockdown group. In addition, NFE2L3 gene knockdown significantly inhibited the migration ability of GC cells, blocked the G0/G1 phase, and increased the proportion of apoptotic cells, which was consistent with the protein expression results. We detected the expression of key proteins that regulate the cell cycle and apoptosis. Downregulation of NFE2L3 promotes the expression of Caspase3 and BAX and inhibits the expression of Bcl2 and CDC2, which is consistent with our experimental results.

Even after active treatment, most patients with GC still die due to tumor recurrence and metastasis [27]. We found that NFE2L3 plays a role in promoting tumor metastasis, which led us to study the mechanism by which NFE2L3 promotes metastasis. EMT plays an important role in the initiation of malignant tumor metastasis [28]. The loss of epithelial characteristics and the acquisition of matrix features reduce cell adhesion and increase cell mobility [29]. Epithelial markers (E-cadherin) were downregulated, and interstitial markers (vimentin and N-cadherin) were upregulated. These molecular changes help us to identify whether the EMT process occurs [30]. In our study, we found the same change trend, which is also consistent with previous research results [15]. In gastric cancer cells with NFE2L3 knockdown, E-cadherin expression was significantly upregulated, whereas vimentin and N-cadherin expression was inhibited. This finding indicates that NFE2L3 may play a role in promoting the EMT in gastric cancer cells.

To further explore the function of NFE2L3, we combined data from public databases to analyze the possible biological functions of NFE2L3 in the body from the point of view of bioinformatics. Increasing evidence indicates that NFE2L2, a protein that is homologous to the NFE2L3 transcription factor, is involved in immune evasion and is associated with immune infiltration of low-grade gliomas [31,32]. We then used TIMER 2.0 (http://timer.comp-genomics.org) to predict the association between NFE2L3 expression and immune cells. We found a correlation between the expression of NFE2L3 and the infiltration of CD4 T and CD8 T cells, suggesting that NFE2L3 expression may be related to immune cell infiltration. GO enrichment analysis showed that NFE2L3 and its related genes were significantly enriched in regulating epidermal cell differentiation and epidermal development, which may be helpful for subsequent treatment with immunotherapy.

However, our research still has some limitations that must be considered. First, the regulatory mechanisms of the human body are very complex, and the phenotype obtained by using gastric cancer cells may not represent those that occur in the body. Second, given the limited experimental conditions, we cannot perform more in-depth research on the regulatory mechanism of NFE2L3, and studies on the correlation between NFE2L3 and immune cells have not been verified at the experimental level. Finally, we may need to establish an animal model to further study the relationship between NFE2L3 and gastric cancer in vivo.

## 5. Conclusion

In conclusion, our research shows that NFE2L3 is highly expressed in GC and promotes GC cell proliferation and metastasis. In addition, silencing NFE2L3 inhibited the cell cycle, increased the proportion of apoptotic cells, and may play a role in promoting cancer through the EMT signaling pathway.

**Table:** 

Group	GO enrichment pathway
BP	*blood coagulation *hemostasis *coagulation *ln utero embryonic development *bile acid biosynthetic process *regulation of epidermal cell differentiation *regulation of epidermis development
CC	*perinuclear endoplasmic reticulum
MF	*DNA-binding transcription activator activity, RNA polymerase ll-specific *DNA-binding transcription repressor activity, RNA polymerase Il-specific *sterol transporter activity *transcription cofactor binding *cholesterol binding *sterol binding *activating transcription factor binding *alcohol binding *steroid binding *RNA polymerase Il distal enhancer sequence-specific DNA binding.

## Highlights

1.This study is the first to focus on the NFE2L3 gene in GC.

2.We verified NFE2L3 expression and function in GC for the first time.

3.We have studied NFE2L3 based on combined experimental and bioinformatics research.
